# The impact of COVID-19 on mortality among diabetic and hypertensive individuals in coastal communities in Bangladesh: evidence from Chakaria health and demographic surveillance system

**DOI:** 10.1186/s12963-026-00493-2

**Published:** 2026-07-13

**Authors:** Srizan Chowdhury, Nahida Hannan Nishat, Ashish Paul, Md Mehedi Hasan, Sayed Saidul Alam, Ammatul Fardousi, Beth A. Tippett Barr, Chodziwadziwa Whiteson Kabudula, Jean Juste Harrisson Bashingwa, Syed Manzoor Ahmed Hanifi

**Affiliations:** 1 Health Systems and Population Studies Division, International Centre for Diarrhoeal Disease Research (icddr,b), Dhaka, Bangladesh; 2 Nyanja Health Research Institute, Salima, Malawi; 3 South African Medical Research Council/Wits Rural Public Health and Health Transitions Research Unit (Agincourt), School of Public Health, Faculty of Health Sciences, University of the Witwatersrand, Johannesburg, South Africa

**Keywords:** HDSS, Chakaria, Excess mortality, Diabetes, Hypertension, COVID-19

## Abstract

**Background:**

COVID-19 was initially considered to be primarily a respiratory illness. However, it soon became evident that individuals with non-communicable diseases (NCDs), particularly Diabetes Mellitus (DM) and Hypertension (HTN), faced a markedly higher risk of severe illness and death. This study assessed excess mortality during the COVID-19 pandemic among individuals living with DM and HTN in rural coastal communities in Bangladesh.

**Methods:**

The study utilised data from the Chakaria Health and Demographic Surveillance System (HDSS), located in the rural sub-district of Chakaria in southeast Bangladesh on the coast of the Bay of Bengal, and operated by the International Centre for Diarrhoeal Disease Research, Bangladesh (icddr,b). Prior to the COVID-19 pandemic, 15,700 individuals were screened for DM and HTN, of whom 8506 aged 18 years or older were included in this study. Crude mortality rates (MR) per 1000 person-years (PYR) were estimated by age, sex, education, and household wealth quintile for individuals with DM/HTN compared to those without, both before and during the pandemic. A Cox proportional hazards model was used to examine differences in mortality risk during the pandemic between individuals with and without DM/HTN, adjusting for sociodemographic factors.

**Results:**

Among the 8506 study participants, 2.7% (n = 232) had DM, 4.8% (n = 407) had HTN, less than 1% (n = 75) had both conditions and 6.6% (n = 564) had either condition. Among individuals without DM/HTN, the crude MR was 7.1 per 1000 PYR overall (6.9 before COVID-19, 7.5 during); among those with DM/HTN, the crude MR was 28.8 overall (21.2 before, 44.5 during). After adjusting for socio-demographic factors, mortality did not increase significantly during COVID-19 among individuals without DM/HTN. However, among those with DM/HTN, adjusted mortality risk during COVID-19 was nearly twice as high as in the pre-COVID-19 period (Hazard Ratio (HR) = 1.89; 95% CI: 1.22–2.91; p < 0.01).

**Conclusion:**

The COVID-19 pandemic significantly increased all-cause mortality among individuals with DM or HTN in the rural sub-district of Chakaria in southeast Bangladesh on the coast of the Bay of Bengal through direct and indirect pathways.

**Supplementary Information:**

The online version contains supplementary material available at 10.1186/s12963-026-00493-2.

## Introduction

Severe acute respiratory syndrome coronavirus 2 (SARS-CoV-2) was first identified in December 2019 in Wuhan City, China, and subsequently evolved into the global Coronavirus disease-19 (COVID-19) pandemic [1]. By August 2023, more than 770 million confirmed cases and 6.9 million deaths had been reported worldwide [2]. Initially, COVID-19 was considered to primarily affect the respiratory system, through inflammation and immunological dysregulation [3]. However, it soon became evident that patients with non-communicable diseases (NCDs), particularly Diabetes Mellitus (DM) and Hypertension (HTN), were at higher risk of infection, severe disease and death [3, 4].

DM and HTN have a synergistic relationship with COVID-19. Early case series from China reported that HTN was present in up to 30% of COVID-19 patients, while other comorbidities were less common [4–6]. Similarly, a study involving 12,594 COVID-19 patients in New York City found that 34.6% had HTN [7]. Moreover, HTN frequently co-occurred among diabetic patients with COVID-19 who experienced severe disease outcomes such as intensive care admission, mechanical ventilation and death [5]. The upregulation of angiotensin-converting enzyme 2 (ACE2) receptor expression induced by certain medications may increase the susceptibility of diabetic patients to SARS-CoV-2 infection. Furthermore, SARS-CoV-2 has been shown to worsen glycemic dysregulation in individuals with DM by promoting systemic inflammation and impairing immune response. DM patients infected with SARS-CoV-2 are at elevated risk of developing severe complications, including thromboembolic events, cardiovascular dysfunction, and respiratory failure [8]. As reported by a Swedish study, individuals with type 2 diabetes had a 40% higher risk of COVID-related hospitalization and intensive care unit admission, and a 1.5 times higher risk of death [9].

Individuals with DM or HTN are more vulnerable to the social and behavioral impacts of public health emergencies such as COVID-19 [10, 11]. Social isolation resulting from physical distancing measures or physical inactivity, and tobacco use [12]. Globally, prevention and treatment services for NCDs were severely disrupted during the COVID-19 pandemic, with particularly severe magnitude in Low- and Middle-Income Countries (LMICs) [13]. A WHO survey covering 155 countries found that patients with comorbid DM or HTN were frequently unable to access healthcare services for their conditions, especially after COVID-19 transitioned from sporadic outbreaks to widespread community transmission [13, 14]. Many people in areas with prolonged lockdowns were unable to access medicines and basic care for chronic conditions [15]. Additionally, outpatient NCD services were compromised as healthcare personnel were reassigned to COVID-19 response efforts [16–18].

As in many other LMICs, the prevalence of DM and HTN is high in Bangladesh. Recent data indicate that HTN affects 19.8% of adults aged 18–69 years in rural areas [19]. In addition, 8.3% of adults have elevated blood glucose based on biomarker measurements, diagnosis, or medication history [19]. Bangladesh also experience an estimated 572,600 (67%) deaths caused by NCDs annually, with 22% occuring prematurely [20]. During the COVID-19 pandemic, over half of the deaths in Bangladesh occurred in individuals with NCDs (52%), with the pandemic contributing to 64.5% of deaths among individuals with DM and 63.8% among those with HTN [21].

The literature on the impact of COVID-19 on populations with comorbid conditions has been enriched by numerous studies carried out across regions and at different points in time, generating context-specific evidence. However, there is a lack of longitudinal studies from LMICs assessing how COVID-19 impacted all-cause mortality among adults living with DM or HTN. This study therefore aimed to estimate excess all-cause mortality among adults with DM/HTN during the Covid-19 pandemic.

## Materials and methods

### Study population

The study population comes from the Chakaria Health and Demographic Surveillance System (HDSS), operated by the International Centre for Diarrhoeal Disease Research, Bangladesh (icddr,b), and located in the rural sub-district of Chakaria in southeast Bangladesh on the coast of the Bay of Bengal. The HDSS monitors a population of approximately 90,000 individuals in 16,000 households across 49 villages. Demographic events occurring within the population, including marriages, pregnancies, births, migrations and deaths, are routinely collected through household visits every three months [22].

### Data collection

#### Health and demographic surveillance system

Data is collected electronically by a team of surveillance workers specially trained for HDSS operations. Two supervisors oversee the regularity and quality of the household visits. The data undergo routine validity and consistency checks by supervisors and data managers. All demographic events necessary to ascertain an individual’s duration of household membership within the HDSS for any given time are recorded. In addition, periodic health surveys are conducted on representative subsets of the HDSS population to supplement the core demographic data.

#### Screening survey for identifying DM/HTN

Between March 4 and April 30, 2016, a survey was conducted to collect information about acute and chronic conditions in a subset of the Chakaria HDSS population. A simple random sample of 3000 households was drawn from the full HDSS household list and interviews were completed in 2955 households. A total of 15,700 members of those households were screened for the presence of DM, HTN and a selected list of other chronic conditions. To improve diagnostic reliability, screening questions included a criterion confirming that any reported condition was made by a registered medical doctor or nurse. Longitudinal data was extracted from the HDSS database for 15,592 individuals. Data on socio-demographic characteristics of the individuals was also extracted to complement the survey dataset.

### Data and variables

**Fig. 1:**
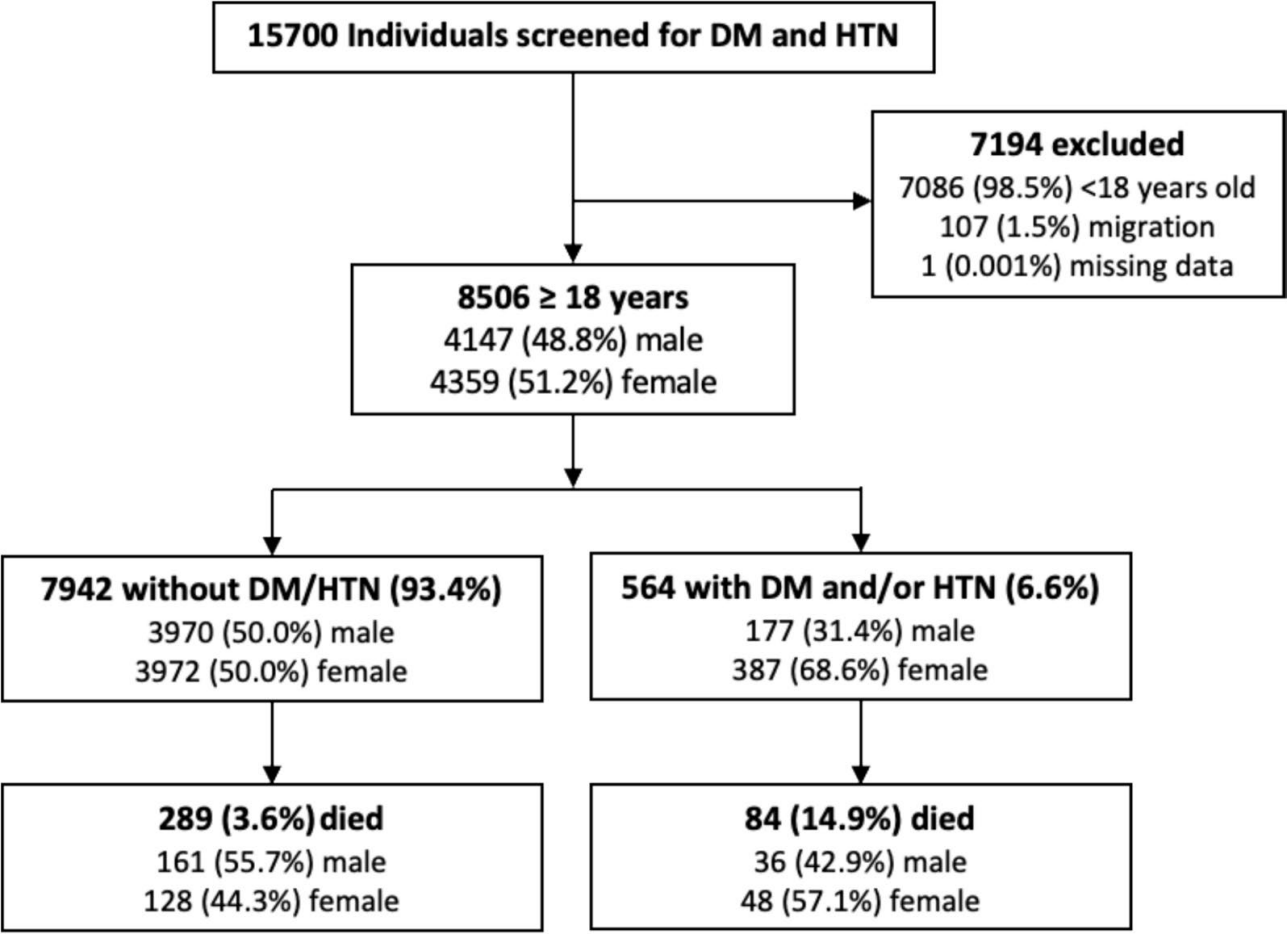
Flowchart of study population

Individuals younger than 18 years at the time of screening (N = 7086) were excluded from the analysis for this study as presented in Fig. 1. For each individual, follow-up began on the screening date and ended at the earliest of: date of death, date of outmigration from the HDSS catchment area, or 31st December, 2021. Each individual’s follow-up time was split by calendar year (starting from 2016 and ending in 2021) and by age group (18–34 years, 35–49 years, 50-64 years, and ≥ 65 years). Follow-up time from 2016 and 2019 was considered as the ‘pre-COVID-19 period’ while 2020 to 2021 was classified as the ‘COVID-19 period’. Individuals identified as having DM or HTN at the start of the follow-up constituted the ‘exposed’ group and those without either condition as the ‘unexposed’ group.

After splitting each individual’s follow-up time, the analytical dataset contained multiple observation records per individual, each linked to a unique individual identification number. Every record was defined by an entry event and an exit event. Entry events included enumeration, birth, transition into a new age group, entry into a calendar year, internal migration within the HDSS area, or migration into the HDSS area from outside. Exit events included death, transition out of an age group, end of a calendar year, internal migration out of a household within the HDSS area, or outmigration from the HDSS area. The primary event of interest was death and all other exit events were treated as censoring. Each record was also linked to a unique household identification number corresponding to the individual’s residence during that period. An individual could migrate out of the HDSS area at any point during the study period and later re-enter the area. The consistency of time ordering for entry and exit events was maintained for each individual. For example, an internal out-migration from one HDSS household was always followed by an internal in-migration into another HDSS household. Person-years of observation were calculated only for periods of residence within the HDSS area, excluding time spent outside the HDSS catchment area.

Socio-economic status was assessed using household data on durable assets. A wealth index was constructed using principal component analysis (PCA) and used to classify households into five wealth quintiles. At the individual level, sex (male or female) and years of formal education (0 years, 1–5 years, and 6 + years) were grouped for the analysis.

### Statistical analysis

Crude mortality rates (MR) per 1000 person-years were calculated for the overall study population and stratified by socio-demographic characteristics. The Kaplan–Meier survival functions were constructed separately for individuals with and without DM/HTN and for the pre-COVID-19 and COVID-19 periods. Cox proportional hazards regression models were then used to estimate the impact of COVID-19 on mortality by DM/HTN status.

In the proportional hazards regression model, the hazard ratio (HR) quantifies the effect of a covariate on the instantaneous rate of the event of interest occurring at any point in time, assuming that the ratio of the rates of events occurring between any two values of any covariate remain constant over time. Specifically, it is the ratio of the hazard rates corresponding to different levels of a covariate. An HR greater than 1 indicates increased risk (e.g., higher mortality), whereas an HR less than 1 indicates reduced risk (e.g., lower mortality) relative to the reference group.

The interaction between ‘period’ and ‘DM/HTN status’ was included in the models to estimate HRs for the COVID-19 versus pre-COVID-19 periods separately for individuals with and without DM/HTN, adjusting for age, education and socio-economic status. The initial model we considered was specified as follows:

$$\begin{aligned}&h(t) = {h}_{0}(t)*\mathrm{e}\mathrm{x}\mathrm{p}({\beta}_{1}*ncdb \\& \quad+ {\beta}_{2}*period + {\beta}_{3}*ncdb*period)\end{aligned}$$

Where, *h(t)* is the hazard function, ${h}_{0}(t)$ is the baseline hazard, *ncdb* is a binary variable indicating if the individual did not have DM/HTN (0) or had DM/HTN (1) and *period* is a binary variable depicting the pre-COVID-19 period (0) and COVID-19 period (1). In this model, the regression coefficient ${\beta}_{1}$ represents the logarithm (log) of the HR for DM/HTN status in the pre-COVID-19 period, ${\beta}_{2}$ represents the log HR during the COVID-19 period versus the pre-COVID-19 period among those without DM/HTN, and ${\beta}_{3}$ represents the interaction effect of DM/HTN status and period.

The above specification of the model does not readily provide an estimate of the HR between pre-COVID-19 period and COVID-19 period for the group with DM/HTN. Therefore, to facilitate interpretation we re-parameterized the regression model as follows:

$$\begin{array}{c}h(t) = {h}_{0}(t)*exp({\alpha}_{1}*ncdb + {\alpha}_{2}*period\\ *(1-ncdb) + {\alpha}_{3}*period*ncdb),\end{array}$$

Where ${\alpha}_{1}$ represents the HR for DM/HTN versus no DM/HTN during the pre-COVID-19 period, ${\alpha}_{2}$ represents the HR for COVID-19 period versus pre-COVID-19 period among individuals without DM/HTN and ${\alpha}_{3}$ represents the HR for COVID-19 period versus pre-COVID-19 period among those with DM/HTN. Confounders were added to the adjusted models in a standard way. This reparameterization of DM/HTN status and period does not affect the estimated coefficients or standard errors for the confounders in the adjusted model. The Wald test was used to evaluate the statistical significance of multiple interaction terms when analysing the subset of individuals with DM/HTN. All analyses were performed using Stata MP (version 17.0) [23].

## Results

Among the 8506 study participants, 2.7% (n = 232) had DM (n = 407), 4.8% (n = 75) had HTN, fewer than 1% (n = 75) had both conditions and 6.6% (n = 564) had either DM or HTN and the remaining participants had neither of the two comorbidities.

Individuals with DM/HTN were predominantly older adults. Among participants aged ≥ 65 years, 17.1% (151/884) had DM/HTN compared to 15.2% (206/1358) of those aged 50–64 years, 6.3% (160/2529) of adults aged 35–49 years and 1.3% (47/3735) of young adults aged 18–34 years. Table 1 summarises the socio-demographic characteristics of the study participants. Household wealth was distributed roughly evenly across quintiles (approximately 20% each), although households in higher wealth quintiles tended to have larger family sizes (data not shown), giving the individual-level distribution a slight skew toward the higher quintiles. This skewness was more pronounced among individuals with DM/HTN, who were disproportionately represented in the highest wealth quintile compared to those without these conditions. Education attainment was also lower among individuals with DM/HTN, with 45.9% of them having no formal education compared to 33.7% of individuals without DM/HTN.

**Table 1:** Background characteristics of the study population at the beginning of follow-up

	**Participants without DM and HTN**	**Participants with DM and/or HTN**	**All participants**
Age			
18–34	3688 (46.4%)	47 (8.3%)	3735 (43.9%)
35–49	2369 (29.8%)	160 (28.4%)	2529 (29.7%)
50–64	1152 (14.5%)	206 (36.5%)	1358 (16.0%)
65 +	733 (9.2%)	151 (26.8%)	884 (10.4%)
Sex			
Male	3970 (50.0%)	177 (31.4%)	4147 (48.8%)
Female	3972 (50.0%)	387 (68.6%)	4359 (51.3%)
Education			
None	2676 (33.7%)	259 (45.9%)	2935 (34.5%)
1–5 years	2211 (27.8%)	143 (25.4%)	2354 (27.7%)
6 + years	3055 (38.5%)	162 (28.7%)	3217 (37.8%)
Wealth quintile			
Lowest	1547 (19.5%)	70 (12.4%)	1617 (19.0%)
Second	1430 (18.0%)	77 (13.7%)	1507 (17.7%)
Middle	1348 (17.0%)	68 (12.1%)	1416 (16.7%)
Fourth	1753 (22.1%)	125 (22.2%)	1878 (22.1%)
Highest	1864 (23.5%)	224 (39.7%)	2088 (24.6%)
All participants	7942 (100%)	564 (100%)	8506 (100%)

**Fig. 2:**
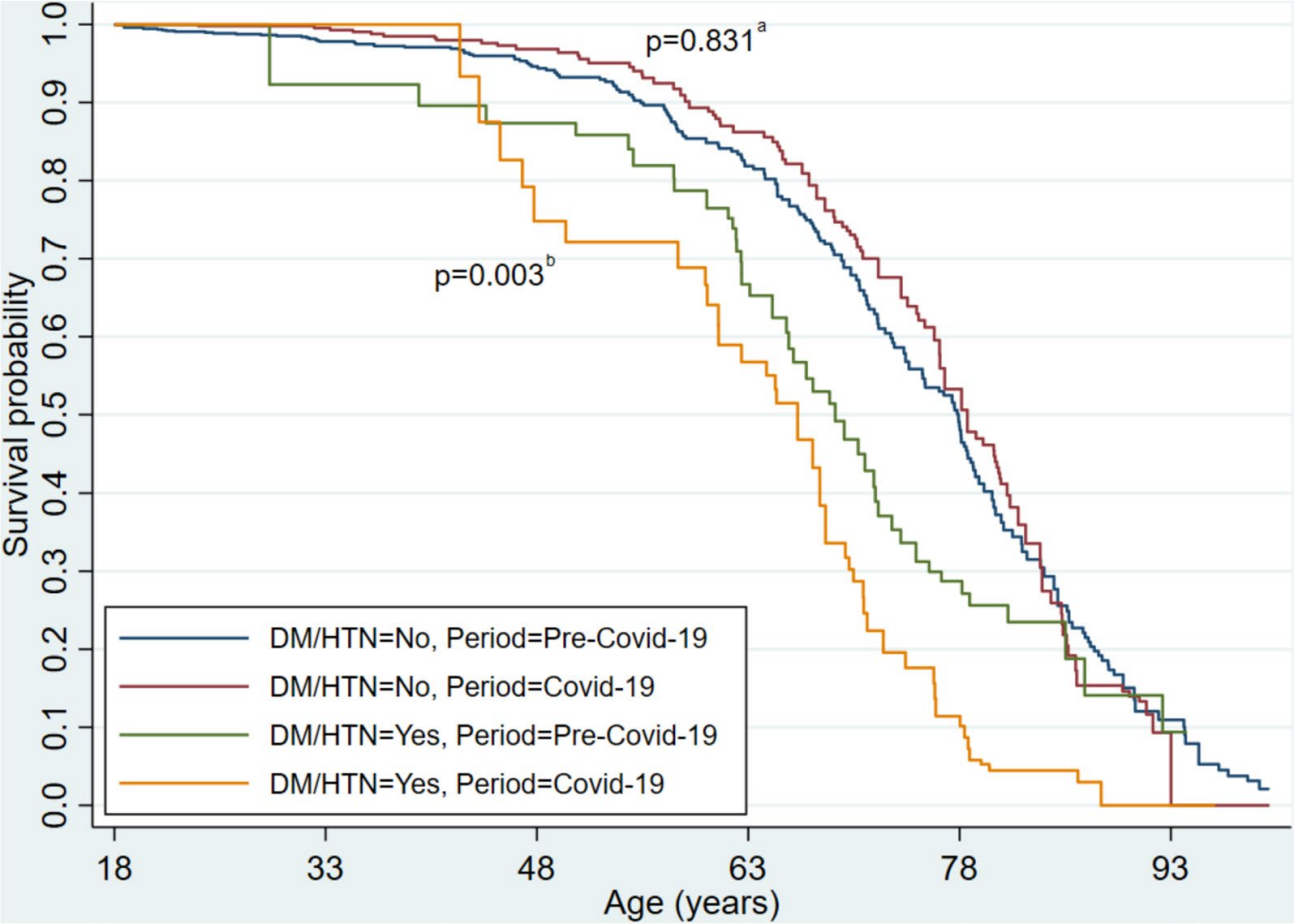
Kaplan Meier estimates of survival probabilities over age as underlying time-scale, segregated by DM/HTN status and period. ^a^The p-value of 0.831 is for the comparison between Pre-COVID-19 and COVID-19 among individuals with no DM/HTN; ^b^the p-value of 0.003 is for the comparison between Pre-COVID-19 and COVID-19 among individuals with DM/HTN

The Kaplan–Meier survivor curves (Fig. 2) show no difference in survival rates before and during the COVID-19 pandemic among individuals without DM/HTN. In contrast, individuals with DM/HTN experienced a statistically significant drop in survival after age of 35 years during the COVID-19 period compared to the pre-COVID-19 period (p = 0.003).

**Table 2:** Crude mortality rate per 1000 person-years by DM/HTN status and period. MR is presented in bold texts wherever significant difference in mortality rate between pre-COVID-19 and COVID-19 is present (p-value < 0.05), where significance is based on the Exact Poisson Test of equality of incidence rates

	**Non-diabetic and non-hypertensive**				**Diabetic or hypertensive**			
	Pre-COVID-19*		COVID-19*		**Pre-COVID-19**		**COVID-19**	
	MR[95% CI]**	**Person-years**	**MR[95% CI]**	**Person-years**	**MR[95% CI]**	**Person-years**	**MR[95% CI]**	**Person-years**
Age (years)								
18–34	1.2[0.8–2.0]	13,786	0.7[0.3–2.0]	5364	4.6[0.7–32.9]	216	0.0[NA]	57
35–49	2.9[1.9–4.3]	7718	1.8[0.9–3.6]	4465	3.2[0.8–12.9]	622	17.8[7.4–42.8]	281
50–64	10.0[7.3–13.7]	3897	8.9[5.7–13.7]	2258	20.6[12.4–34.2]	727	23.4[12.2–45.0]	384
65 +	53.4[44.4–64.3]	2096	56.3[44.3–71.6]	1189	**58.3[39.0–86.9]**	412	**126.2[87.2–182.8]**	222
Sex								
Male	7.4[6.1–9.0]	13,718	8.9[6.9–11.5]	6623	31.3[19.9–49.0]	608	60.1[37.4–96.7]	283
Female	6.4[5.2–7.9]	13,779	6.0[4.4–8.2]	6653	**16.8[11.2–25.3]**	1369	**37.8[25.6–56.0]**	661
Education (years)								
None	14.2[12.0–16.8]	9507	13.2[10.3–17.0]	4679	**24.3[16.0–37.0]**	904	**55.8[37.4–83.3]**	430
1–5 years	3.7[2.5–5.3]	7670	3.7[2.2–6.3]	3757	**17.6[9.2–33.9]**	511	**59.6[35.3–100.6]**	235
6 + years	2.6[1.8–3.8]	10,319	4.8[3.2–7.2]	4840	19.6[10.8–35.3]	562	14.3[5.4–38.2]	279
Wealth quintile								
Lowest	7.7[5.6–10.5]	5085	8.5[5.5–13.2]	2342	18.4[6.9–49.1]	217	40.1[15.0–106.7]	100
Second	6.0[4.2–8.5]	5022	7.2[4.6–11.3]	2635	30.8[15.4–61.6]	260	46.4[20.9–103.3]	129
Middle	6.6[4.6–9.4]	4692	6.9[4.2–11.2]	2333	**16.6[6.2–44.3]**	240	**68.6[34.3–137.1]**	117
Fourth	6.5[4.8–8.9]	6261	8.2[5.5–12.2]	2935	32.7[19.7–54.2]	459	50.6[28.0–91.4]	217
Highest	7.6[5.8–10.1]	6438	6.6[4.3–10.2]	3031	**13.7[7.6–24.8]**	801	**34.2[19.8–58.8]**	381
Overall	6.9[6.0–8.0]	27,497	7.5[6.1–9.1]	13,276	**21.2[15.7–28.7]**	1977	**44.5[32.9–60.2]**	944

*Pre-COVID-19 refers to the years 2016–2019, and COVID-19 refers to 2020–2021

**MR is presented as mortality rates per 1000 person-years with 95% confidence intervals

The crude MR among individuals without DM/HTN was 7.1 deaths per 1000 PYR overall (during pre-COVID-19 and COVID-19 periods combined) compared with 28.8 deaths per 1000 PYR among individuals with DM/HTN. Among individuals without DM/HTN, the crude MR was 6.9 deaths per 1000 person-years (PYR) before the COVID-19 pandemic and 7.5 deaths per 1000 PYR during the COVID-19 pandemic (Table 2). In contrast, among individuals with DM/HTN, the crude MR was 21.2 deaths per 1000 PYR before COVID-19 and 44.5 deaths per 1000 PYR during COVID-19 (Table 2).

Mortality patterns across socio-demographic strata are summarized in Table 2. Among individuals without DM/HTN, age-specific MR showed little variation between pre-COVID-19 and COVID-19: 1.2 deaths per 1000 PYR (pre-COVID-19) vs. 0.7 deaths per 1000 PYR (during COVID-19) for age 18–34 years, 2.9 deaths per 1000 PYR vs. 1.8 deaths per 1000 PYR for age 35–49 years, 10.0 deaths per 1000 PYR vs. 8.9 deaths per 1000 PYR for age 50–64 years, and 53.4 deaths per 1000 PYR vs. 56.3 deaths per 1000 PYR for age 65 years and over. Among individuals with DM/HTN, those aged ≥ 35 years experienced substantial increase in age-specific MR between pre-COVID-19 and COVID-19 periods: MR increased from 3.2 deaths per 1000 PYR to 17.8 deaths per 1000 PYR for age 35–49 years, 20.6 deaths per 1000 PYR to 23.4 deaths per 1000 PYR for age 50–64 years, and 58.3 deaths per 1000 PYR to 126.2 deaths per 1000 PYR for age 65 years and over.

Sex differences in mortality were evident among the participants. Among individuals with DM/HTN, males had a higher crude MR compared to the females. The crude MR increased from 16.8 to 37.8 deaths per 1000 PYR for females with DM/HTN and from 31.3 to 60.1 deaths per 1000 PYR for males with DM/HTN during COVID-19. Household wealth gradients were minimal among individuals without DM/HTN. The crude MR varied between 6.0 and 8.5 deaths per 1000 PYR across wealth quintiles over the two periods. In contrast, among individuals with DM/HTN, crude MR ranged from 13.7 to 32.7 deaths per 1000 PYR before COVID-19 and nearly doubled in each household wealth quintile during COVID-19. Additionally, individuals with no education consistently experienced higher mortality compared to those with one or more years of education.

**Fig. 3:**
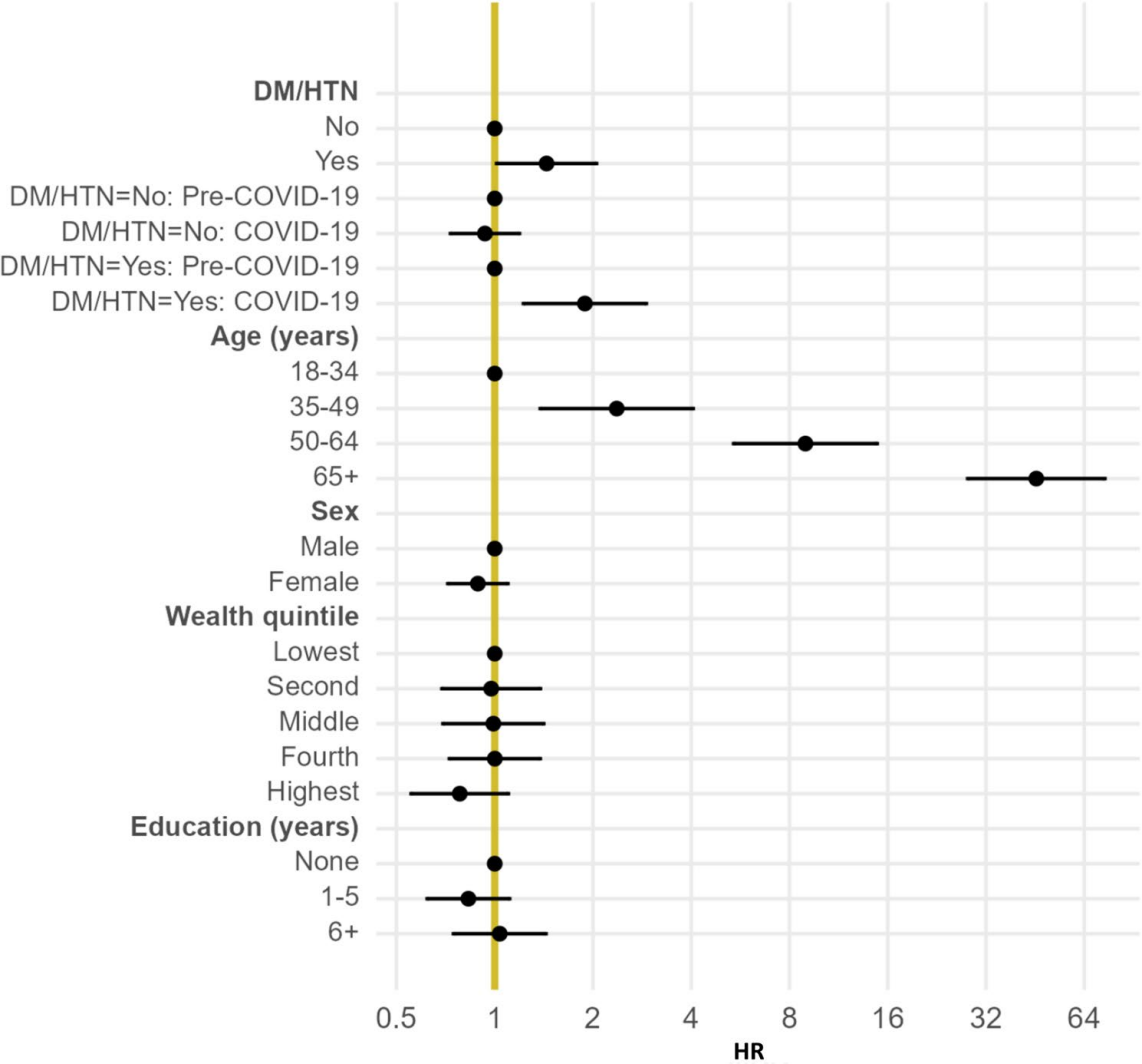
Forest plot showing adjusted HR for DM/HTN status, period, interaction between DM/HTN status and period, and all the confounding factors

Survival analysis of the data showed that prior to the COVID-19 pandemic individuals with DM or HTN had a 44% (HR = 1.44; 95% CI: 1.01–2.05, p = 0.042) higher rate of all-cause mortality compared to those without these conditions, adjusting for age and other socio-demographic factors (Fig. 3 and Additional file 1). Among individuals without DM/HTN, the adjusted MR remained stable between the pre-COVID-19 and COVID-19 periods (HR = 0.93; 95% CI: 0.73–1.19, p = 0.579). In contrast, for individuals with DM/HTN, the adjusted MR increased by 89% during COVID-19 relative to the level during the pre-COVID-19 period (HR = 1.89; 95% CI: 1.22–2.91, p = 0.004).

Age was the strongest predictor of mortality. The adjusted MR for individuals aged 35–49 years was 2.36 times (95% CI: 1.38–4.06) that of individuals aged 18–34 years, while individuals aged 65 + years had 45.63 times (95% CI: 28.11–74.06) the adjusted MR of individuals aged 18–34 years. Females had lower mortality than males although this was not statistically significant after adjusting for age (p-value = 0.272). Belonging to lower wealth quintile was also not significantly associated with increased mortality (HR highest vs. lowest quintile = 0.78; 95% CI: 0.55–1.10, p = 0.159). While unadjusted analysis suggested differences in mortality by education level, these effects disappeared after adjusting for other factors.

Further analysis of participants with DM/HTN assessed whether excess mortality during COVID-19 varied by age, sex, level of education and socio-economic status. Only a single death was observed in this subgroup among individuals aged 18–34 years. The increase in mortality from the pre-COVID-19 period to the COVID-19 period was not significantly different across age groups 35–49, 50–64 and 65 + years (relative to change in mortality between periods for age-group 35–49 years, HR for interaction between age-group 50–64 years and COVID-19 period = 0.21, 95% CI: 0.03–1.29, p = 0.091, HR for interaction between age-group 65 + years and COVID-19 period = 0.39, 95% CI: 0.07–2.21, p = 0.288; joint Wald test for both interaction terms to be zero: Chi-squared statistic = 3.34, p = 0.188). No significant difference was also observed between men and women in increase in mortality from 2016–2019 to 2020–2021 (relative to change in mortality between periods for males, HR for interaction between female sex and COVID-19 period = 1.08, 95% CI: 0.45–2.57, p = 0.870). Similarly, no differential change in mortality was observed from pre-COVID-19 period to COVID-19 period by level of education (relative to change in mortality between periods for no education-group, HR for interaction between education-group 1–5 years and COVID-19 period = 1.59, 95% CI: 0.56–4.52, p = 0.383, HR for interaction between education-group 6 + years and COVID-19 period = 0.30, 95% CI: 0.08–1.08, p = 0.066; joint Wald test for both interaction terms to be zero: Chi-squared statistic = 5.26, p = 0.072) and socio-economic status (relative to change in mortality between periods for lowest wealth quintile, HR for interaction between second wealth quintile and COVID-19 period = 0.48, 95% CI: 0.08–3.03, p = 0.438, HR for interaction between middle wealth quintile and COVID-19 period = 1.42, 95% CI: 0.21–9.70, p = 0.718, HR for interaction between fourth wealth quintile and COVID-19 period = 0.52, 95% CI: 0.10–2.81, p = 0.448, HR for interaction between highest wealth quintile and COVID-19 period = 0.86, 95% CI: 0.16–4.68, p = 0.857; joint Wald test for all interaction terms to be zero: Chi-squared statistic = 2.81, p = 0.590).

## Discussion

### Main findings

The population affected by either of the two major comorbidities, DM or HTN, experienced a substantially higher mortality during the COVID-19 period compared with both the pre-COVID-19 period and the rest of the population in Chakaria, a rural resource-poor setting in Bangladesh. Although the pandemic did not significantly affect overall mortality in the entire population, it significantly elevated all-cause mortality among individuals with DM or HTN, likely through direct and indirect pathways.

Age was a significant predictor of mortality in the present study. Individuals aged 35–49 years had a mortality rate 2.4 times higher than those aged 18–34 years, while those aged 65 years and above experienced a mortality rate 45.6 times higher than those in the 18–34 year age group. This finding is consistent with several other studies that reported age as a key determinant of COVID-19-related severity and mortality risk [24–27].

### Interpretation

Individuals with DM and HTN develop upregulation of the ACE2 receptor, which facilitates COVID-19 infection [28]. Studies indicate that treating HTN patients with ACE inhibitors to reduce blood pressure increases ACE2 expression. This is concerning because the COVID-19 virus uses ACE2 for human cell entry [29, 30]. The ACE2 receptor triggers a cytokine storm which exacerbate comorbidities in the host through an inflammatory immune response [31]. The bidirectional link between DM and COVID-19 is well established. DM is a major risk factor for severe SARS-CoV-2 infection, intensive care unit admission, and death [9, 32, 33], while SARS-CoV-2 infection itself contributes to insulin resistance and deteriorating glycemic control via complex pathophysiological mechanisms [34, 35]. During the pandemic, the stay-at-home mandates led to reduced physical activity, increased TV viewing time, poorer dietary and eating habits and increased psychological stress, all of which exacerbate metabolic disorders such as DM and increase the likelihood of COVID-19-related hospitalization [36]. Furthermore, DM-related outpatient visits and laboratory monitoring declined significantly, reflecting disruptions in healthcare delivery for DM management [37, 38]. Research has shown that mortality rates associated with DM and HTN remained elevated during the pandemic years from 2020 to 2022, even after excluding deaths directly attributed to COVID-19. This persistence suggests that interactions between these chronic conditions and pandemic-related factors such as healthcare system strain and socio-economic disruptions contributed to excess mortality [39].

Bangladesh experienced multiple waves of COVID-19 beginning in 2020, during which government-imposed movement restrictions led to limited access to routine healthcare [40, 41]. These disruptions likely contributed to increased all-cause mortality during the COVID-19 period. Assessing excess mortality provides a valuable means of capturing both the direct and indirect impacts COVID-19. Importantly, not all excess deaths were caused by COVID-19 infection itself. Some may have been caused by the indirect effects of restricted services and strained healthcare systems during the COVID-19 period, which could have increased NCD-related mortality as reported by other studies [15, 42, 43].

### Strengths

A representative subset of the population residing in the study area was selected comprising individuals with and without DM/HTN to preserve population-level prevalence of DM/HTN. Individuals with DM/HTN selected for the follow-up had been diagnosed by registered medical doctors or nurses. The study leveraged an established HDSS, well-suited for conducting longitudinal research, to obtain reliable socio-economic and demographic data and to accurately measure follow-up time and mortality outcomes for each individual over a period of four years prior to COVID-19 and two years during the pandemic.

### Limitations

The study did not incorporate into the analysis the severity of DM or HTN at the start of follow-up, changes in these conditions over time, the emergence of new cases, or the presence of additional comorbidities among the study participants. The study only assessed all-cause mortality among the participants, which limit the ability to distinguish between the direct and indirect pathways through which COVID-19 could have elevated mortality among individuals with DM/HTN compared to the rest of the population. The study sample size was insufficient to explore more complex interactions involving age, sex, and existing comorbidities. Moreover, behavioral risk factors (e.g., diet and physical activity), external factors (e.g., access to treatment), and broader social determinants, all of which could have affected the management of comorbidities, were not included in the analysis. Potential changes in these factors before and during the COVID-19 pandemic, which may have moderated the impact of COVID-19 on mortality risk, were also not assessed. Future studies, designed with a priori power considerations and more comprehensive data, are needed to explore these relationships in greater depth.

## Conclusion

The COVID-19 pandemic highlighted both the heightened vulnerability of people living with comorbidities such as DM and HTN and the inadequacy of health systems and infrastructures, particularly in low-resource settings, to provide adequate care during sudden public health emergencies. COVID-19 increased the risk of dying among individuals with comorbidities, either through direct infections or by disrupting health services, care-seeking behavior and healthy lifestyles, particularly in the most remote and underserved communities. In light of these findings, this study supports calls for implementation of proactive public health strategies aimed at reducing the prevalence of chronic conditions like DM and HTN and improving their management. Strengthening such initiatives is essential not only for mitigating the impact of future pandemics but also for improving overall population health outcomes.

## Supplementary Information

Supplementary material 1.

## Data Availability

Data for computing mortality indicators by year, age and sex are available from the MRC/Wits Agincourt Research Unit Data Repository (https://data.agincourt.co.za/index.php/catalog/335). Data containing other covariates used in the analysis reported in this manuscript can be accessed through a formal request to the corresponding author.

## Abbreviations

COVID-19: Coronavirus disease 2019
SARS-CoV-2: Severe acute respiratory syndrome coronavirus 2
NCD: Non-communicable diseases
HDSS: Health and demographic surveillance systems
icddr,b: International centre for diarrhoeal disease research, bangladesh
DM: Diabetes mellitus
HTN: Hypertension
MR: Mortality rate
HR: Hazard ratio
PYR: Person-year
ACE-2: Angiotensin-converting enzyme 2
LMIC: Low- and middle-income country
