# Supplementary material for: The impact of COVID-19 on mortality among diabetic and hypertensive individuals in coastal communities in Bangladesh: evidence from chakaria health and demographic surveillance system
Source: Popul Health Metr. 2026 Jul 13;23(Suppl 2):79. doi: 10.1186/s12963-026-00493-2 (PMC13421514; doi:10.1186/s12963-026-00493-2)
Supplement: Supplementary file 1 — Supplementary material 1. [file 12963_2026_493_MOESM1_ESM.docx]

**Table S1**. Crude and adjusted hazard ratios according to diabetes and hypertension (DM/HTN) status, period, interaction of DM/HTN status and period and other confounding factors

|  | **Unadjusted** | | **Adjusted** | |
| --- | --- | --- | --- | --- |
|  | **HR(95% CI)** | **p-value** | **HR(95% CI)** | **p-value** |
| DM/HTN |  |  |  |  |
| No | Ref. |  | Ref. |  |
| Yes | **3.07(2.20-4.30)** | **<0.001** | **1.44(1.01-2.05)** | **0.042** |
| DM/HTN=No: period |  |  |  |  |
| Pre-COVID-19 | Ref. |  | Ref. |  |
| COVID-19 | 1.07(0.84-1.37) | 0.569 | 0.93(0.73-1.19) | 0.579 |
| DM/HTN=Yes: period |  |  |  |  |
| Pre-COVID-19 | Ref. |  | Ref. |  |
| COVID-19 | **2.09(1.36-3.20)** | **0.001** | **1.89(1.22-2.91)** | **0.004** |
| Age (years) |  |  |  |  |
| 18-34 | Ref. |  | Ref. |  |
| 35-49 | **2.49(1.47-4.22)** | **0.001** | **2.36(1.38-4.06)** | **0.002** |
| 50-64 | **10.06(6.29-16.10)** | **<0.001** | **8.95(5.39-14.85)** | **<0.001** |
| 65+ | **51.98(33.59-80.43)** | **<0.001** | **45.63(28.11-74.06)** | **<0.001** |
| Sex |  |  |  |  |
| Male | Ref. |  | Ref. |  |
| Female | 0.84(0.69-1.03) | 0.102 | 0.89(0.72-1.1) | 0.272 |
| Wealth quintile |  |  |  |  |
| Lowest | Ref. |  | Ref. |  |
| Second | 0.90(0.64-1.27) | 0.560 | 0.98(0.69-1.38) | 0.888 |
| Middle | 0.92(0.65-1.31) | 0.648 | 0.99(0.69-1.41) | 0.957 |
| Fourth | 1.06(0.78-1.46) | 0.699 | 1.00(0.73-1.38) | 0.996 |
| Highest | 1.01(0.74-1.38) | 0.962 | 0.78(0.55-1.10) | 0.159 |
| Education (years) |  |  |  |  |
| 0 | Ref. |  | Ref. |  |
| 1-5 | **0.34(0.26-0.45)** | **<0.001** | 0.83(0.62-1.11) | 0.211 |
| 6+ | **0.26(0.20-0.34)** | **<0.001** | 1.04(0.75-1.44) | 0.832 |
